# Comparison of Four Purification Methods on Serum Extracellular Vesicle Recovery, Size Distribution, and Proteomics

**DOI:** 10.3390/proteomes11030023

**Published:** 2023-07-25

**Authors:** Dianny Elizabeth Jimenez, Muhammad Tahir, Muhammad Faheem, Wellington Bruno dos Santos Alves, Barbara de Lucena Correa, Gabriel Rocha de Andrade, Martin R. Larsen, Getulio Pereira de Oliveira, Rinaldo Wellerson Pereira

**Affiliations:** 1 Programa de Pós-Graduação em Ciências Genômicas e Biotecnologia, Universidade Católica de Brasília, Brasília 71966-700, Brazil; elizabethjp24@gmail.com (D.E.J.); faheem08@live.com (M.F.); welligtonnetrix@hotmail.com (W.B.d.S.A.); baabicorrea@hotmail.com (B.d.L.C.);; 2 Department of Biochemistry & Molecular Biology, University of Southern Denmark, 5230 Odense, Denmark; tahir.bio@gmail.com (M.T.); mrl@bmb.sdu.dk (M.R.L.); 3 Department of Biomedical Sciences, University of North Dakota School of Medicine & Health Sciences, Grand Forks, ND 58202, USA; 4 The Barnett Institute for Chemical & Biological Analysis, Northeastern University, Boston, MA 02115, USA

**Keywords:** extracellular vesicle, EVs, exosome, microvesicle, purification methods, molecular profiling, mass spectrometry, proteomics

## Abstract

In recent decades, the role played by extracellular vesicles in physiological and pathological processes has attracted attention. Extracellular vesicles are released by different types of cells and carry molecules that could become biomarkers for the diagnosis of diseases. Extracellular vesicles are also moldable tools for the controlled release of bioactive substances in clinical and therapeutic applications. However, one of the significant challenges when studying these exciting and versatile vesicles is the purification process, which presents significant difficulties in terms of lack of purity, yield, and reproducibility, reflected in unreliable data. Therefore, our objective in the present study was to compare the proteomic profile of serum-derived EVs purified using ExoQuick™ (Systems Biosciences), Total Isolation Kit (Life Technologies), Ultracentrifugation, and Ultrafiltration. Each technique utilized for purification has shown different concentrations and populations of purified particles. The results showed marked differences in distribution, size, and protein content, demonstrating the need to develop reproducible and reliable protocols to isolate extracellular vesicles for their clinical application.

## 1. Introduction

The term “extracellular vesicles” (EVs) refers to a broad range of cellular membrane layered structures delivered directly from the cell membrane or through the endosomal pathway [1], named microvesicles (100–1000 nm) and exosomes (50–150 nm), respectively [2]. Extracellular vesicles are released to the extracellular environment from various cells, such as lymphocytes, dendritic cells, macrophages, mast cells, neurons, Schwann cells, oligodendrocytes, epithelial cells, and numerous tumor cells [3,4]. EVs have also been isolated from various body fluids, such as blood, urine, saliva, amniotic fluid, pleural fluid, semen, and breast milk [5,6]. They are also isolated from conditioned cell culture medium from every kind of cell investigated [7]. EVs’ roles as mediators of cell-to-cell communication in pathological and physiological processes, stimulating cell membrane receptors [8], or delivering mainly nucleic acids and proteins have been widely demonstrated, continuously increasing the research community’s interest in them [9].

The increasing interest in EVs has demanded the implementation of less laborious methods than standard ultracentrifugation [10]. Polymer-based precipitation [11] and ultrafiltration [12] are among the most widespread substitutes for ultracentrifugation [13].

**Figure 1:**
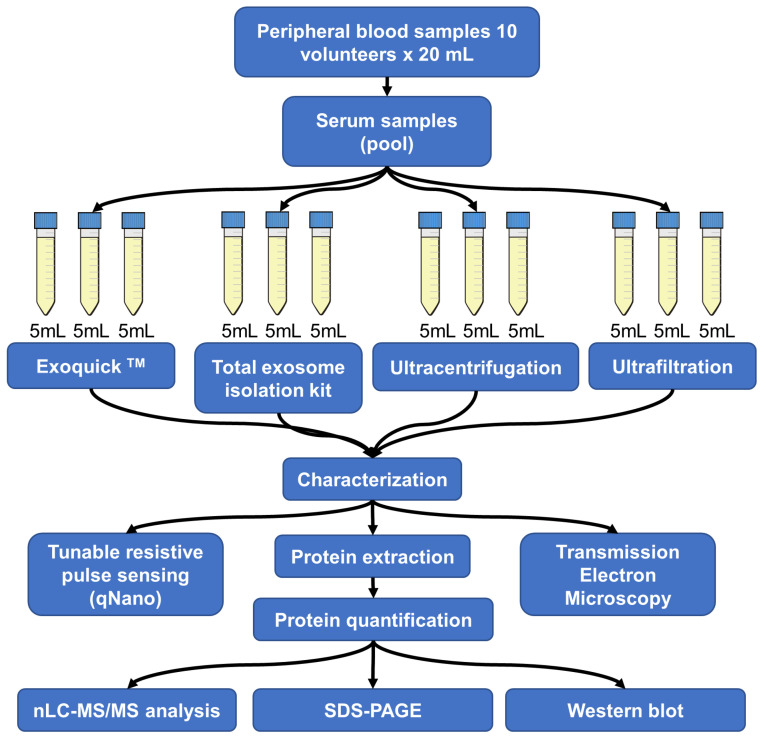
The workflow of EV isolation by four different techniques from human serum. The total volume of each of the ten serum samples was pooled. The pooled serum sample was used in each EV purification method and was carried out in technical triplicates.

Despite the purification method used, the knowledge of its recovery rate and purity of EVs are essential to the downstream investigation of their roles in physiology and physiopathology, or as biomarkers [14]. Here, we show that four broadly used EV purification methods (ExoQuickTM-Systems Biosciences, Total isolation kit-Life Technologies, Ultracentrifugation, and Ultrafiltration; Figure 1) deliver different recovery rates, size distributions, and proteomic profiles in EVs purified from human serum samples.

## 2. Material and Methods

### 2.1. Sample Collection

Ten healthy volunteers (five males and five females—ages ranging from 20 to 35 years) donated 20 mL of peripheral blood samples. All subjects signed a written informed consent document approved by the Universidade Católica de Brasília IRB under 08984012.6.0000.0029 protocol. Each blood sample was collected by venipuncture of the medial ulnar vein using sterile vacuum syringes (Vacutainer). The blood samples were left to rest for 10 min before the serum fraction was transferred and centrifuged at 3000 rpm for 10 min. Five milliliters of pooled serum sample was used in each EV purification method.

### 2.2. Extracellular Vesicle Isolation Techniques

#### 2.2.1. ExoQuick Precipitation (EX)

Polymer-based ExoQuick™ precipitation was carried out according to the manufacturer’s instructions (System Biosciences Inc., Mountain View, CA, USA). Five mL of pooled serum sample was centrifuged at 3000× *g* for 15 min to remove cellular debris. Then, the supernatant was transferred to a sterile tube, and 1 mL of ExoQuick™ (Exosome Precipitation Solution) was added. Next, the sample was refrigerated at 4 °C overnight (12 h) and then centrifuged at 1500× *g* for 30 min at 4 °C until the beige-like pellet was formed at the bottom of the tube. Finally, the supernatant was removed, and the pellet was resuspended in 500 μL of PBS buffer and stored at −80 °C for further analysis.

#### 2.2.2. Total Isolation Kit (KI)

Polymer Total Exosome Isolation Kit precipitation was carried out according to the manufacturer’s instructions (Life Technologies, Carlsbad CA, USA). A total volume of 5 mL of serum was collected from the pool of samples and centrifuged at 2000× *g* for 30 min, following the manufacturer’s protocol, to remove cell debris. Next, the supernatant was transferred to a new tube, and 1 mL of Total Isolation Kit reagent was added. The mixture was vortexed and resuspended by pipetting until a homogenous sample was obtained and then incubated at 4 °C for 30 min. After incubation, the sample was centrifuged at 10,000× *g* for 10 min at room temperature. Finally, the supernatant was discarded, and the pellet containing the extracellular vesicles was resuspended in 500 μL of PBS buffer and stored at −80 °C for further analysis.

#### 2.2.3. Ultracentrifugation (UC)

From the pooled serum sample, 5 mL was submitted to three steps of centrifugation starting at 300× *g* for 5 min, going to 2000× *g* for 10 min, and finishing with 16,500× *g* for 20 min at 4 °C. This procedure was applied to remove cell debris. Next, the supernatant from the last centrifugation step was filtered over a 0.22 μm filter to remove particles larger than 200 nm. The filtrate was ultracentrifuged at 118,000× *g* for 70 min at 4 °C. The pellet was resuspended in a PBS buffer. A subsequent washing was performed in the ultracentrifuge tubes by adding PBS, and it was centrifuged again at 118,000× *g* for 70 min at 4 °C [15,16]. The pellet was resuspended in 500 μL of PBS buffer and then at −80 °C for further experiments.

#### 2.2.4. Ultrafiltration (UF)

From the pooled sample, 5 mL of serum was added to Amicon Ultra-15 Centrifugal Filter Units^®^ (Merck Millipore, Darmstadt, Germany) and centrifuged at 4000× *g* for 10 min until a volume of 500 μL was obtained. Then, the sample was stored at −80 °C for further analysis.

### 2.3. Characterization of Extracellular Vesicles

#### 2.3.1. Determination of Size Distribution by Tunable Resistive Pulse Sensing (TRPS)

The TRPS method was performed on a Qnano^®^ instrument (Izon Science, Burnside, New Zealand). The manufacturer’s suggested calibrator, composed of CP100 polystyrene particles diluted in PBS, was used. The nanopores used were NP150 (extension of 85 nm to 300 nm) and NP100 (extension of 70 nm to 200 nm). The parameters established and configured for the reading of samples were 7 Pa positive pressure, 45 nm aperture, and a voltage of 0.80 V. For the readings, 40 μL of each of the samples was applied. The minimum reading acceptable for each sample was 500 particles per min.

#### 2.3.2. Protein Extraction and Sample Preparation for MS

Aliquots of 500 μL from each purification technique were collected and conditioned in a protease inhibitor cocktail (Sigma-Aldrich, San Luis, MO, USA) microtubes to posterior protein extraction. Subsequently, the samples were sonicated in a water bath three times for 5 min with vortex mixing in between [17]. Positive control of the extraction and protein quantification was performed with the preparation of mononuclear cells from serum. An aliquot of 200 μL of PBS and 2 μL of protease inhibitor cocktail (PIC) were used as a negative control. Protein quantification was performed by the absorbance method with a NanoDrop 2000 c (Thermo Fisher Scientific Inc, Waltham, MA). Approximately 100 μg of the protein sample from each replicate of the four conditions was solubilized in a 2 M urea and 6 M thiourea lysis buffer. The sample was reduced with 10 mM DTT for 30 min at room temperature and alkylated for 40 min, followed by 20 mM iodoacetamide in the dark at room temperature. Subsequently, the proteins were digested with 0.04 AU Lys-C (Wako, Japan) for 3 h at 28 °C. Following Lys-C digestion, the solution was diluted eight times with 50 mM TEAB buffer, and trypsin was added (50:1 (*w*/*w*) protein:trypsin) overnight at 28 °C with constant shaking. As described previously, the resulting tryptic peptides were desalted using homemade microcolumns of Poros Oligo R2/R3 packed resin [18]. The purified peptides were quantified using a Biochrome 30 amino acid composition analyzer (Cambridge, UK) as described in [18].

#### 2.3.3. nLC-MS/MS Analysis of Tryptic Peptides

A total of 1 μg of the purified peptides was applied to a Proxeon EASY-nLC system (Thermo Fisher Scientific, Odense, Denmark) connected to a Q Exactive HF (Thermo Fisher Scientific) mass spectrometer. Mobile phases were as follows: (A) 0.1% FA in water; and (B) 95% ACN, 0.1% FA in water. The peptides were loaded into an 18 cm in-house packed reversed-phase capillary column (75 μm ID) packed with ReproSil-Pur C18 AQ 3 μm material in solution A. Peptides were eluted into the mass spectrometer using 134 min gradients as follows: 1–3% B in 3 min, 3–25% B in 100 min, 25–45% B in 20 min, 45–100% in 3 min, and 100–100% 8 min at a flow rate of 250 nanoliters/min. Data-dependent acquisition mode (top 20) was used for the MS method. The parameters used for MS were as follows: whole MS scan mass area of 400–1600 m/z, resolution—120,000, maximum injection time was set to 100 ms, the automatic gain control (AGC) target for full MS was 3 × 10^6^, and the dynamic exclusion was set to 40 s. For the isolation of precursor ions, an m/z window of 1.2 was used, followed by fragmentation using higher-energy collision dissociation (HCD) with normalized collision energy (NCE) of 28. A 30,000 resolution was used to measure fragment ions with a maximum injection time of 100 ms and AGC target value of 1 × 10^3^ during MS/MS.

#### 2.3.4. Proteomics Data Analysis of MS/MS

Proteome Discoverer version 1.4.0.288 (Thermo Fisher Scientific) was used to process the raw files. A database search was performed against the SwissProt human database (version 2017.01) using Mascot as a search engine. The search parameters were kept as follows: trypsin as digestion enzyme with two missed cleavage sites, precursor mass tolerance of 10 ppm, carbamidomethylation of cysteines as a fixed modification, and methionine oxidation as a dynamic modification. A Venn diagram was made from the lists of the identified proteins to see the overlap between different extracellular vesicle isolation methods. Enzyme prediction analysis for the identified proteins was performed using the UniProt database. Cellular component and molecular function gene ontologies were performed using FUNRICH 3.0 [19]. The mass spectrometry proteomics data were deposited to the ProteomeXchange via MassIVE partner repository [20] with the database identifier PXD016667.

#### 2.3.5. SDS-PAGE and Western Blot Analysis

A total of 20 μg of protein samples obtained from different techniques were denatured at 95 °C for 5 min and separated on a 10% polyacrylamide gel (Bio-Rad Laboratories) using PBS as the buffer dilution used for immunoblotting. The SDS-PAGE broad range marker (Bio-Rad) was used as a parameter. After transfer into PVDF membranes in transfer buffer (25 mM Tris-base, 192 mM glycine, 20% methanol (vol/vol), and 0.1% SDS), blocking was performed for 3 h in blocking solution (5% skimmed milk in TBST) at room temperature with slow and constant stirring. Membranes were incubated in 10 mL of the primary antibody (anti-CD9, anti-CD63, anticalnexin) overnight (12 h), and the samples were washed with TBST. Subsequently, they were incubated in 10 mL of secondary antibody conjugated with rabbit peroxidase for one hour at room temperature with slow and constant agitation. After the second incubation, the samples were washed with TBST and placed in a revelation buffer.

#### 2.3.6. Transmission Electron Microscopy (TEM)

The samples were processed through the negative contrast technique with uranyl acetate. A sheet was submerged in formvar (0.6–1.0%) for a few sec and then dried on filter paper. The sheet was submerged in water to remove the formed film. Subsequently, the grids were placed in contact with the formvar and dried overnight. The grids were cut and fixed inside a petri dish, and 3 μL of contrast agent and uranyl acetate were added. After drying, the samples were observed under a microscope.

### 2.4. Statistical Analysis

EV concentration, size, and protein concentration were compared among different purification methods (ExoQuickTM, Total Isolation Kit, Ultrafiltration, and Ultracentrifugation) using a one-way Kruskal–Wallis with Dunn test for multiple comparisons of groups. *p* value was considered significant if <0.05. Statistical analyses were performed using GraphPad Prism 8.0 (GraphPad Software, San Diego, CA, USA).

## 3. Results

### 3.1. The Size Distribution of the Extracellular Vesicles

A comparative study was carried out on extracellular vesicle samples purified by different techniques (ExoQuickTM, Total Isolation Kit, Ultrafiltration, and Ultracentrifugation), obtaining differences in the concentrations of particles per milliliter of the sample and the size in nanometers. The samples were measured with technical replicates (triplicate) in Qnano by the verification of the particle concentration in NP100 and NP150 nanopores, allowing us to ascertain how reproducible the readings are through each purification protocol. The samples were processed at a constant temperature, stored at −80 °C, and thawed on ice for further analysis. Two nanopore sizes were used for the measurement, NP100, and NP150, to cover a wider variety of particle sizes. Maximum pore aperture parameters of 45 mm, pressure of 7 Pa, and voltage of 0.8 V were maintained.

**Figure 2:**
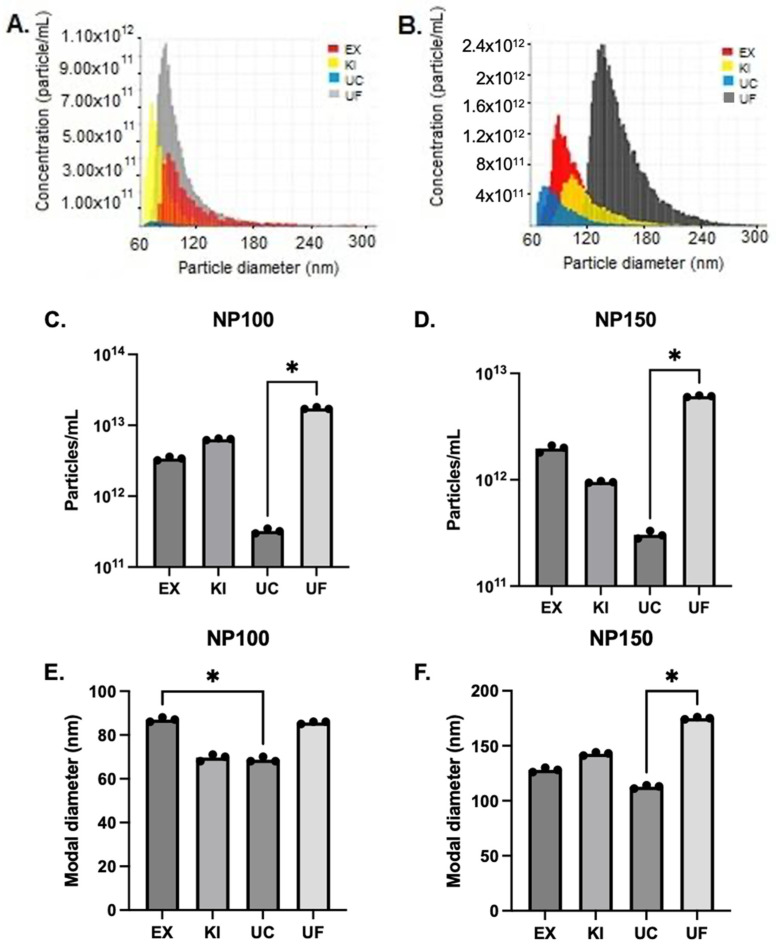
Comparison of size distribution and concentration of extracellular vesicles purified by four different techniques: (EX) ExoQuick^TM^, (KI) Total Isolation Kit, (UC) Ultracentrifugation, and (UF) Ultrafiltration. (**A**) Concentration and size distribution of extracellular vesicles measured by tunable resistive pulse sensing (qNano) using a Nanopore NP100, and (**B**) and Nanopore NP150. (**C**) Reproducibility of the four techniques according to the concentration of particles measured by tunable resistive pulse sensing (TRPS) using a Nanopore NP100, (**D**) and Nanopore NP150. (**E**) Modal particle diameter of extracellular vesicles measured by tunable resistive pulse sensing (qNano) using a Nanopore NP100, (**F**) and Nanopore NP150. The data in this graph are the values of each technical replicate. Asterisks represent *p* ≤ 0.05 using Dunn’s multiple comparison test.

There were differences in the size distribution and recovery of particles for each purification technique. In all purification techniques, more particles were obtained in nanopore NP100 than in nanopore NP150. The ultrafiltration technique presented the highest median values of particle concentration for the two nanopores NP100 and NP150, 1.7 × 10^13^ ± 4.7 × 10^11^ and 6.1 × 10^12^ ± 8.2 × 10^10^, respectively (Figure 2A,B).

The amount of the recovered particles differs significantly between the two polymer purification techniques, with ExoQuickTM achieving the maximum recovery for NP100 with median values for a particle concentration of 3.4 × 10^12^ ± 1.6 × 10^11^ and Total Isolation Kit (KI) obtaining a higher concentration of particles compared to ExoQuickTM (EX) with median values of 6.4 × 10^12^ ± 1.2 × 10^11^ for NP100.

The ultracentrifugation technique recovered the lowest concentration of particles compared to all other techniques, with median values for extracellular microvesicle modal diameters of 69 ± 0.9 nm and 113 ± 1.2 nm for nanopores NP100 and NP150, respectively, and with median particle concentrations of 3.2 × 10^11^ ± 2.1 × 10^10^ using NP100 and 3.0 × 10^11^ ± 2.1 × 10^10^ with NP150 (Supplementary Table S1). Kruskal-Wallis was performed to test for significant differences in particle concentration and modal diameter for each of the processed samples. The results obtained in technical replicates from the four extracellular vesicle purification techniques present significant differences (*p* < 0.05). When comparing the different techniques, no significant difference in the particle concentration of EVs was observed (*p* > 0.05), except when comparing the concentration of EVs isolated using UC and UF (*p* < 0.05) (Figure 2C,D). For size distribution, we found a statistically significant difference in EVs isolated by ExoQuickTM compared to UC using nanopore NP100, and UC to ultrafiltration using nanopore NP150 (*p* < 0.05) (Figure 2E,F).

The distribution shows that most particles passed through the pores with approximately the same blocking duration. In the technical replicate, most of the particles or microvesicles are monodispersed as the data points are regularly concentrated in the same region, making the protocols of the techniques reproducible in our experiment. The area of particle concentration consists of a blocking duration of up to 10 ms and 20 ms. Blocking events that are outside the area are assumed to be aggregates or larger species of particles that are present in the solution. Being the same size with different blocking times indicates that the blocking events detected are not single-particle events.

### 3.2. Total Protein Concentration from Extracellular Particles Purified Using Four Different Techniques

**Figure 3:**
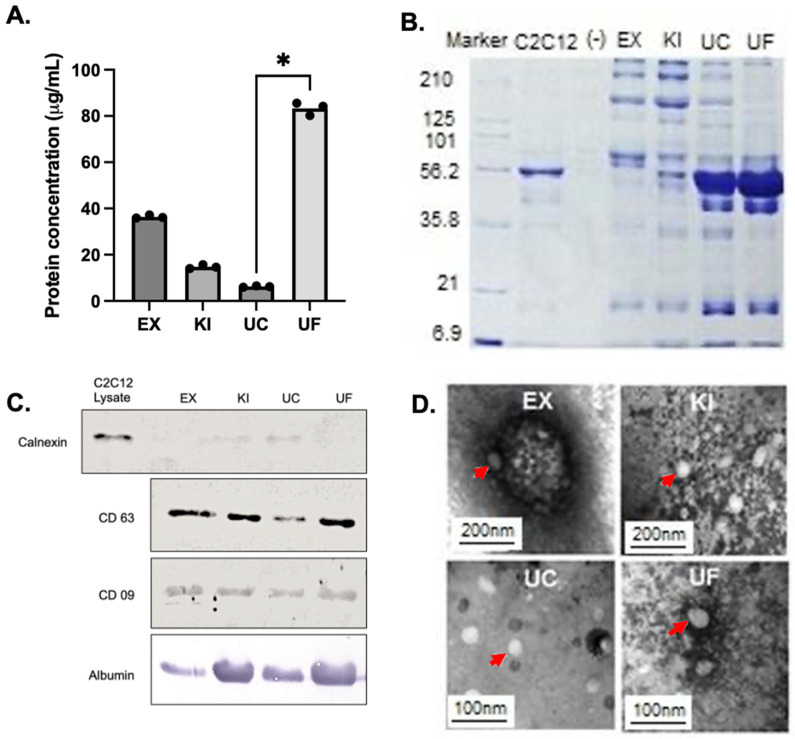
(**A**) Quantification of total exosomal proteins per mL of the purification techniques. The data in this graph are the values of each technical replicate. Asterisks represent *p* ≤ 0.05 using Dunn’s multiple comparison test. (**B**) Protein profile of the purification techniques analyzed by 12% SDS-PAGE: (Marker) Protein marker (Bio-Rad), (C2C12 EVs) positive control, (−) Negative control, (EX) ExoQuick^TM^, (KI) Total Isolation Kit, (UC) Ultracentrifugation, and (UF) Ultrafiltration. (**C**) Western blot analysis detected an antibody against the exosomal surface protein CD9 and an antibody against the exosomal surface protein CD63 and albumin. Calnexin was used to show the absence of endoplasmic reticulum proteins in purified EVs. (**D**) Negative staining of EVs by transmission electron microscopy (TEM). Red arrows indicate particles with the size and shape characteristics of EVs.

Extracellular vesicle protein concentration data revealed differences for each purification method used, with the highest level of protein detected in ultrafiltration with median values of 83,321 μg/mL and the lowest in ultracentrifugation with 6254 μg/mL (Figure 3A). The proteins obtained after purification were analyzed by SDS-PAGE. The results showed a prominent 60 kDa band in the samples, corresponding to the molecular weight of the most abundant protein in the blood, albumin, which was confirmed by Western blotting (Figure 3B).

### 3.3. EV-Specific Protein Markers Investigated by Western Blotting

Western blotting revealed the detection of the calnexin protein in the positive control (polymorphonuclear cells) in the 90–100 kDa band. However, no calnexin was detected in the extracellular vesicles derived by any of the four techniques. On the other hand, the detection of tetraspanins CD9 (band 20–30 kDa) and CD63 (band 26 kDa) verified the presence of extracellular microvesicles in the samples purified by the different techniques. Finally, the detection of albumin (band 60 kDa) in the samples shows that it was the primary contaminant (Figure 3C).

### 3.4. Transmission Electron Microscopy Characterization of the Purified Extracellular Vesicles

A difference in the morphology of the purified extracellular vesicles with different techniques showed a size variation between 50 nm and 200 nm when observed with transmission electron microscopy (TEM) (Figure 3D).

### 3.5. Mass Spectrometry Protein Identification

**Figure 4:**
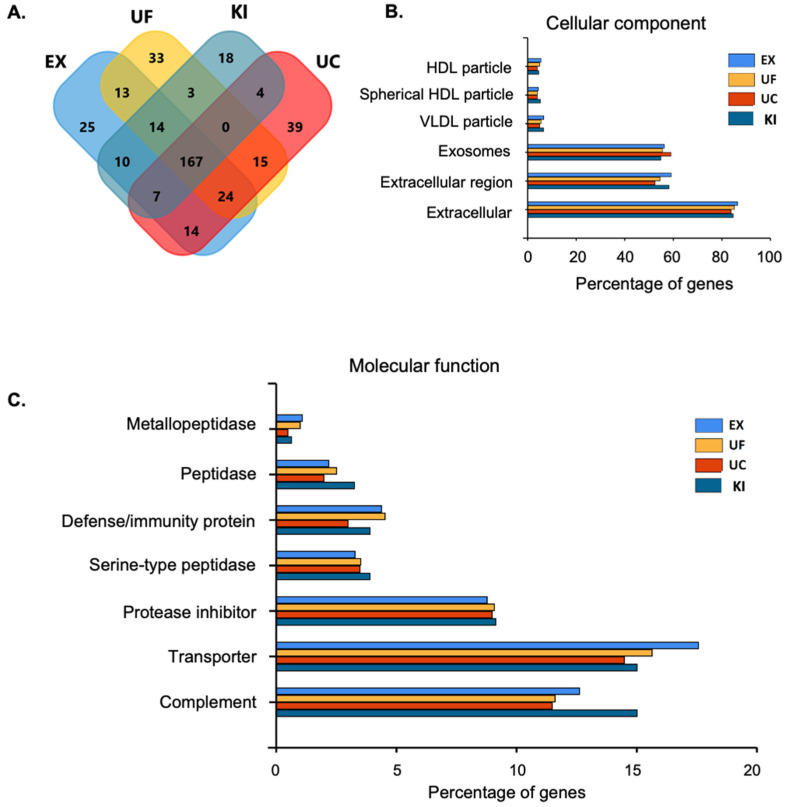
(**A**) A Venn diagram of the unique and overlapping identified proteins from the extracellular vesicles isolated by using four different approaches, (EX) ExoQuick^TM^, (KI) Total Isolation Kit, (UC) Ultracentrifugation, and (UF) Ultrafiltration. (**B**) Cellular component analysis. (**C**) Molecular function analysis.

After the isolation of the extracellular vesicles by using four different methods, proteins were extracted and digested with trypsin, and LC-MSMS analyzed the resulting peptides. The Venn diagram shows overlapping and unique proteins identified among all the EV isolation methods (Figure 4A)

The gene ontology (GO) analysis of the cellular location confirmed that most of the proteins identified belonged to exosomes or were extracellular (Figure 4B). In contrast, the molecular function analysis revealed transport activity for the enriched proteins (Figure 4C).

**Figure 5:**
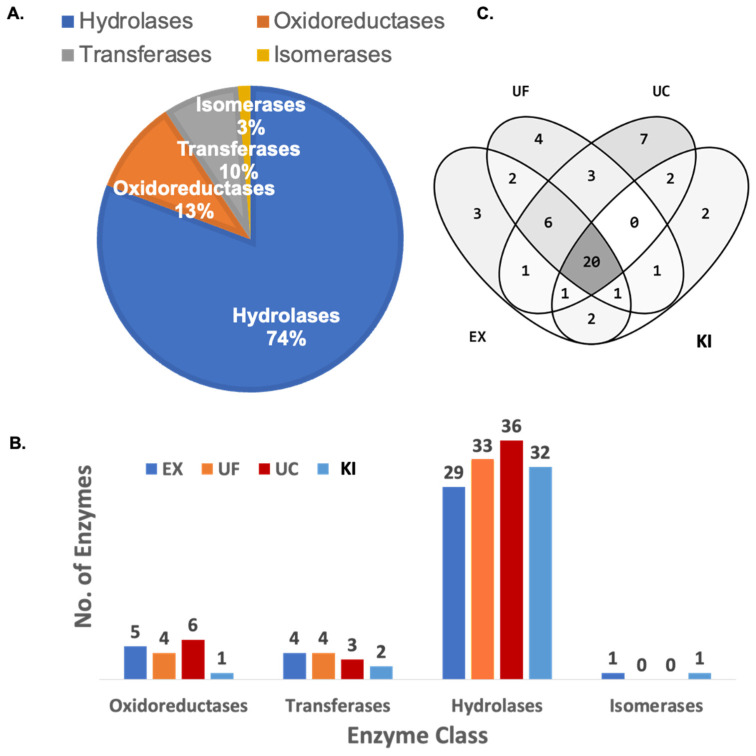
Predicted enzyme profile of the extracellular vesicles identified proteins from all four methods, (EX) ExoQuick^TM^, (KI) Total Isolation Kit, (UC) Ultracentrifugation, and (UF) Ultrafiltration. (**A**) Pie chart distribution of the enzyme classes. (**B**) Number of enzymes identified for each enzyme class. (**C**) A Venn diagram of the predicted enzymes showing the unique and overlapping characteristics of the different isolation methods.

The enzyme prediction analysis for the identified extracellular vesicle proteins was performed using the UniProt database, and 74% of the enzymes were hydrolases, 13% oxidoreductases, 10% transferases, and 3% isomerases (Figure 5A). The total number of enzymes identified in each class of enzymes is shown in Figure 5B. The number of overlapping and unique enzymes identified among all the EV isolation methods is represented in the Venn diagram (Figure 5C) A complete list of the identified proteins and predicted enzymes can be found in Supplementary Table S2.

## 4. Discussion

The rapid advances in science and technology have facilitated the development of techniques for isolating extracellular vesicles in appreciable quantities. Each of them explores a particular parameter, such as density, shape, size, and the existence of surface proteins [15,21]. The presence of extracellular vesicles readily available in body fluids such as urine, saliva, and blood could be an alternative to avoid the use of invasive modes in clinical use [22]. Currently, extracellular vesicles represent potential biomarkers for diagnosis, are sources of treatment, and are crucial components in cellular communication [23].

To facilitate the study and application of these extracellular vesicles, they must be isolated, specifically from cells and interfering components [24]. However, a good choice and a conscious analysis of the methodology used to recover extracellular vesicles from these different biological fluids are required [13].

Therefore, the present work is focused on evaluating four different techniques for isolating extracellular vesicles from human serum samples, comparing them in terms of particle size, distribution, and analysis of protein content.

Particle concentration and size distribution were shown to be different among the four extracellular vesicle techniques investigated. Among the investigated techniques, ultrafiltration retrieved the highest number of particles with median values of 1.7 × 10^13^ ± 4.7 × 10^11^ (NP100) and 6.1 × 10^12^ ± 8.2 × 10^10^ (NP150). Lower values were obtained using ultracentrifugation with an approximately ten times lower number of particles than ultrafiltration.

Difficulties in the process of ultracentrifugation, such as the recovery of non-visible precipitates, make it challenging to resuspend the final pellet, contributing to experience loss in the recovery of extracellular vesicles [15,25].

The results of the number of extracellular vesicles obtained could also be related to differences in processing times and temperatures of the samples during each purification technique [26]. Exoquick^TM^ and Total Isolation Kit take a processing time ranging from 50 min to 60 min. Ultrafiltration techniques require 4 h to 6 h, while ultracentrifugation requires 6 h to 8 h.

The presence of non-extracellular vesicle particles or protein aggregates was also observed by some researchers in purification by using commercial polymer precipitation systems [27]. The technique-based differences found in the extracellular vesicle count and size distribution illustrate the need for technologies to discriminate the type of particle isolated during purification and the detection of copurified aggregates [6].

In this study, particles outside the monodispersed zone were observed by TRPS in all the techniques, indicating different charges and, hence, different natures of the particles. These particles may be protein aggregates formed between the completion of the purification and the storage stage of the sample. Although all the samples were subjected to a prefiltration process using a 0.22 μm filter membrane, sizes exceeding 220 nm were observed in the particle distribution.

The extracellular vesicles acquire surface electrical charge in a polar medium. This charge capacity of the extracellular vesicles can affect the ionization of surface groups of the membrane and differential ion adsorption from the electrolyte solution [28]. Therefore, pH variation can affect the membrane ionic potential of extracellular vesicles, causing their aggregation. The extracellular vesicles’ stability depends on the medium’s zeta potential, ionic strength, and pH. The zeta potential increases with increasing pH; thus, a higher zeta potential leads to higher electrostatic repulsion and, ultimately, reduces aggregation tendency [28,29]. In this sense, studies of the time of aggregation of extracellular vesicles and the use of different dilution buffers are necessary to establish efficient protocols for characterization.

Another relevant data point in the protein profile was the identification by SDS-PAGE gel bands with sizes between 50 kDa and 60 kDa, which could represent proteins with molecular weights comparable with albumin, which is not part of the conventional extracellular vesicle proteome [30,31]. These bands were more prominent in samples purified by Total Isolation Kit (KI) and ultrafiltration than in the other methods. Other comparative studies of extracellular vesicle purification techniques, such as ultracentrifugation, density gradient, and immunocapture using serum and plasma, have also shown the presence of albumin during purification [10]. Albumin sticks to the membranes of extracellular vesicles and contaminates purification [32]. Since albumin is the primary plasma-soluble protein and a predominant component of the isolates, ultracentrifugation would not be an ideal method to obtain a sufficient and pure preparation of extracellular vesicles, i.e., as needed for in vivo experiments or for analytical assays of proteomics or RNA analysis. However, extracellular vesicles isolated by this technique may be appropriate for analysis where contaminant materials do not interfere with measurements [10].

When comparing the morphology of the purified extracellular vesicles using transmission electron microscopy, we found that the ultrafiltration presents rounded and better-delimited extracellular vesicles, which could suggest the preservation of its integrity compared to the other techniques. It does not present any type of aggregation related to contaminants or even the same grouped extracellular vesicles due to the purification method used [33].

Since electron microscopy is performed with procedures that require fixation and dehydration steps, there is likely to be a reduction in size and changes in the morphology of the extracellular vesicles. However, this technique has been widely used for such detection [34,35].

EVs contain a complex mixture of proteins, lipids, and nucleic acids that can vary depending on the cell type of origin, the physiological state of the cell, and the method of isolation. It is estimated that EVs can contain thousands of different proteins, some of which are common to all EVs and others that are specific to certain cell types or physiological conditions [1]. Furthermore, the proteome of EVs can include various proteoforms, which are different forms of the same protein resulting from post-translational modifications (PTMs), alternative splicing, or genetic variations. PTMs, such as phosphorylation, acetylation, or glycosylation, can alter the function and localization of proteins and can thus have significant biological implications [36].

In this study, the proteomic profile revealed 386 proteins in the whole serum (Supplementary Table S2). The cellular component GO analysis showed that most proteins identified were exosomal and lipoproteins. Lipoproteins are present in extracellular vesicles in mammals and are related to the structural organization of the membrane [37].

The presence of enzymes within EVs suggests that these vesicles can actively modulate the biochemical environment of recipient cells by delivering functional enzymes. The exact composition and function of enzymes within EVs can vary depending on the cell type, physiological state, and cellular signaling context. Understanding the cargo of enzymes within EVs can provide insights into their biological functions and potential roles in disease processes. When comparing the MS of each purification technique, 167 of 386 proteins were found in all techniques. Hydrolase enzymes of the protease, glycosylase, and lipase types represented 74% of the enzymes analyzed using the UniProt database. The remaining enzymes were recognized as 10% transferases, 13% oxidoreductases, and 3% isomerases. Hydrolase enzymes could probably be related to microvesicle release processes by cellular stress stimuli [38,39].

The Hsp70 protein chaperone was also found in our analysis. This protein is involved in the presentation of antigens and is ubiquitously expressed in all living organisms. Its location is ordinarily cytoplasmic and nuclear, although it is also secreted outside and is increased by cellular stress [40,41].

In general, the proteins identified in the extracellular vesicles analyzed correspond mainly to components of the cytoplasm, membrane, wall, ribosome, mitochondria, nucleus, and extracellular components. These proteins might be located inside the extracellular vesicles, through the membrane, or attached to the membrane surface [3,42,43].

Ultrafiltration with low-speed differential centrifugation seems to be one of the most efficient techniques in terms of time and cost compared to other techniques analyzed since it requires conventional centrifuges and little processing time. It also allows good recovery of extracellular vesicles by conserving their integrity. Thus, methods based on filtration may be an alternative on a large scale for recovering extracellular vesicles from cell culture supernatants or biological fluids having high volumes.

However, since the contamination level with non-EV proteins in the Ultrafiltration group is high, this method may not be optimal for the analysis of low-abundance proteins from EV. The choice of EV isolation method should be based on the specific research objectives, available resources, and the desired purity and yield of EVs. Researchers often combine multiple isolation techniques or use additional purification steps to enhance the reliability and specificity of EV isolation.

Our results show that basic extracellular vesicle research and the question of its therapeutic potential require efficient and reproducible isolation methods. No purification method discriminates between extracellular vesicles, exact amounts, morphologies, or protein compositions. The methodologies presented are not yet sufficient to determine with certainty the type of population recovered. Therefore, the need arises to establish identification criteria that allow efficient characterization and the creation of reproducible and reliable protocols.

## Data Availability

All data generated or analyzed during this study are included in this article and in the supplementary information files.

## Supplementary Materials

The following supporting information can be downloaded at: https://www.mdpi.com/article/10.3390/proteomes11030023/s1, Table S1. The size distribution of the extracellular vesicles. Particle concentration and mode values for modal diameter obtained for exosome preparations isolated from serum, using ExoQuickTM (Systems Biosciences), Total Isolation Kit (Life Technologies), Ultracentrifugation and Ultrafiltration; Table S2. A complete list of the identified proteins and predicted enzymes.
