# Supplementary material for: Comparison of Four Purification Methods on Serum Extracellular Vesicle Recovery, Size Distribution, and Proteomics
Source: Proteomes. 2023 Jul 25;11(3):23. doi: 10.3390/proteomes11030023 (PMC10443378; doi:10.3390/proteomes11030023)
Supplement: Supplementary file 1 [file proteomes-11-00023-s001.zip › Supplementary table S1.pdf]

Supplementary material

		EX	KI	UC	UF
**NP 100**	Mode (nm)	87 ± 0.8	70 ± 1.2	68 ± 0.9	86 ± 0.5
	Concentration (particles/ml)	$3.4x10^{12}$ ± $1.6x10^{11}$	$6.4x10^{12}$ ± $1.2x10^{11}$	$3.2x10^{11}$ ± $2.1x10^{10}$	$1.7x10^{13}$ ± $4.7x10^{11}$
**NP 150**	Mode (nm)	128 ± 1.6	143 ± 1.2	113 ± 1.2	175 ± 0.8
	Concentration (particles/ml)	$2.0x10^{12}$ ± $1.2x10^{11}$	$9.5x10^{11}$ ± $1.2x10^{10}$	$3.0x10^{11}$ ± $2.1x10^{10}$	$6.1x10^{12}$ ± $8.2x10^{10}$

Table 1. The size distribution of the extracellular vesicles. Particle concentration and mode values for modal diameter obtained for exosome preparations isolated from serum, using ExoQuick™ (Systems Biosciences), Total Isolation Kit (Life Technologies), Ultracentrifugation and Ultrafiltration.
